# Adult cancer-related hemophagocytic lymphohistiocytosis – a challenging diagnosis: a case report

**DOI:** 10.1186/s13256-017-1344-x

**Published:** 2017-06-27

**Authors:** Michael A. Hust, Boris R. A. Blechacz, Diana L. Bonilla, Naval Daver, Cristhiam M. Rojas-Hernandez

**Affiliations:** 1 Department of Internal Medicine, The University of Texas Health Science Center, Houston, TX USA; 2 Department of Gastroenterology, Hepatology and Nutrition, The University of Texas MD Anderson Cancer Center, Houston, TX USA; 3 Department of Immunology, The University of Texas MD Anderson Cancer Center, Houston, TX USA; 4 Department of Leukemia, The University of Texas MD Anderson Cancer Center, Houston, TX USA; 5 Section of Benign Hematology, The University of Texas MD Anderson Cancer Center, 1515 Holcombe Blvd. Unit Number: 1464, Houston, TX 77030 USA

**Keywords:** Hemophagocytosis, Cancer, Diagnosis, Adult

## Abstract

**Background:**

Adult hemophagocytic lymphohistiocytosis is a secondary immunopathologic phenomenon, mainly secondary to malignancy, infection, or autoimmune disorders.

The performance of diagnostic criteria, studied in the pediatric population, is yet to be validated in the adult population. Some of the criteria include cytopenias and organomegaly that are inherent features to malignant processes, thus making the diagnosis of hemophagocytic lymphohistiocytosis a challenge in patients with cancer.

**Case presentation:**

We describe the case of a 54-year-old white man with history of metastatic maxillary sinus adenoid cystic carcinoma who had severe liver injury and cytopenias with progressive clinical deterioration. We performed an evaluation, by flow cytometry, of the expression of surface markers in his natural killer cells that revealed remarkable abnormalities. His syndrome eventually fulfilled criteria for hemophagocytic lymphohistiocytosis and he received therapy with steroids with interval clinical improvement. Unfortunately, he refused further cytotoxic treatment and died 2 weeks later.

**Conclusions:**

The conventional criteria for the diagnosis of hemophagocytic lymphohistiocytosis are suboptimal for adult patients with cancer resulting in delays in diagnosis and timely initiation of treatment. The diagnostic criteria have to be re-evaluated in patients with cancer; novel, easily available, and accurate diagnostic methods are needed.

## Background

Hemophagocytic lymphohistiocytosis (HLH) is a life-threatening immunopathologic syndrome with increasing prevalence in adults, necessitating systematic characterization and development of adult-specific criteria. Early recognition and diagnosis is critical with <10% survival without prompt treatment [1]. The HLH-2004 criteria offer a basis for clinical consideration, yet lack validation in the adult population [2]. Criteria such as cytopenias and organomegaly are inherent to malignant processes, especially hematologic or metastatic disease or concomitant chemotherapy.

We present a case of malignancy-related HLH with clinical manifestations that represented diagnostic ambiguity and difficulty in therapy initiation. We also describe the potential use of the evaluation of surface markers in cytokine-producing natural killer (NK) cells and cytotoxic NK cells by flow cytometry for diagnosis of that entity. Finally, we synthetize a literature review exploring current and developing HLH diagnostic criteria, as well as barriers to therapy, in particular for malignancy-related HLH.

## Case presentation

A 54-year-old white man with a past medical history of right maxillary sinus adenoid cystic carcinoma with metastatic lesions to lung and vertebrae, hypertension, hypothyroidism, and nonalcoholic steatohepatitis presented to our emergency room in June 2016 with mental status changes accompanied by abdominal pain, dyspnea, and fever. He was not on active therapy for his malignancy. He previously received a trial regimen of coenzyme Q10, 12 g every 3 days, from October 2015 to April 2016. His past medical history was remarkable for two prior episodes of ascites and 3 months of progressive liver dysfunction and coagulopathy (Table 1). He had atorvastatin-induced myalgias leading to its discontinuation in April 2016 although his creatine kinase levels were not elevated. He was taking increasing doses of acetaminophen (2 grams daily) prior to admission. His physical examination vital signs were stable with one recorded febrile episode. In general, he appeared ill and lethargic, and was only oriented to person. He exhibited diminished bibasilar breath sounds, ascites with guarding, hypoactive bowel sounds, right lower quadrant tenderness, and hepatosplenomegaly.

**Table 1:** Laboratory and diagnostic data

Characteristic (reference laboratory value)	Baseline	Peak of liver enzyme abnormalities	Admission	Steroid initiation	1 week post-steroid	2 weeks post-admission
White blood cell (4–11×10^3^/uL)	5.5	4.0	5.0	8.5	4.8	41.0
Hemoglobin (14.0–18.0 gm/dL)	11.0	9.0	11.1	10.8	9.5	13.1
Platelet (140–440 K/uL)	164	109	60	75	29	96
Prothrombin time (12.7–15.0 seconds)	15.7	20.6	22.5	35.7	24.6	26.3
International normalization ratio (0.90–1.20)	1.25	1.80	2.02	3.76	2.22	2.43
Partial thromboplastin time (24.7–35.9 seconds)	42.0	37.7	31.6	45.1	30.1	32.9
Fibrinogen (mg/dL)	452		166	141	173	155
Albumin (g/dL)	2.9	2.3	2.3	2.2	3.3	4.0
Aspartate aminotransferase (15–46 U/L)	150	807	395	314	136	145
Alanine aminotransferase (7–56 U/L)	67	319	54	53	26	39
Alkaline phosphatase (38–126 U/L)	123	139	432	350	182	187
Gamma-glutamyl transferase (8–78 U/L)	42			148	291	252
Serum lactate dehydrogenase (U/L)	2372	3879	3449		2769	3867
Ferritin (30–400 ng/mL)		28,942		10,283	6031	5863
Serum triglyceride level (≤1500 mg/dL)	167	212	101			
NK-cell activity (7–125 LU30)						5
CD25 (<1033 pg/mL)				3910		
IL-1 (<1.0 pg/mL)						1.8
IL-6 (<5.0 pg/mL)						27
IFN-y (<2.0 pg/mL)						<0.4
Tumor necrosis factor (1.2–15.3 pg/mL)						20.9

*IFN* interferon, *IL* interleukin, *LU* lytic unit, *NK* natural killer

Laboratory data were remarkable for hyperferritinemia, hypofibrinogenemia, anemia, and thrombocytopenia along with elevated transaminases and coagulopathy (Table 1). A peripheral blood smear showed neutrophilia, monocytosis, and reticulocytopenia. No microangiopathic changes were seen. Extensive platelet clumping was noted.

Imaging studies revealed small pleural effusions, ascites, and hepatosplenomegaly with no evidence of portal hypertension or splanchnic thrombosis. We were suspicious of HLH in light of laboratory and physical examination findings. Additional differential diagnosis workup – infectious, autoimmune, acetaminophen levels – yielded unremarkable results, including: serology for hepatitis A virus (HAV), hepatitis B virus (HBV), hepatitis C virus (HCV), hepatitis E virus (HEV), cytomegalovirus (CMV), Epstein–Barr virus (EBV), herpes simplex virus (HSV), alpha 1-antitrypsin levels, and antinuclear, anti-mitochondrial, anti-smooth muscle, and transglutaminase antibodies (immunoglobulin A (IgA) and immunoglobulin G (IgG)). ADAMTS 13 activity was >34%. HLH-specific laboratory studies were sent, including soluble CD25 (sCD25), NK cell activity studies, and bone marrow biopsy.

He continued to deteriorate with multiple organ failure including renal failure, myocardial injury, and respiratory failure requiring intubation. Empiric therapy, considering the evidence of liver injury and the possibility of HLH, with N-acetylcysteine on a 20-hour intravenous protocol and dexamethasone 8 mg intravenously administered three times daily was initiated. HLH chemotherapy was not done during this time as hepatotoxicity risk outweighed benefits and a definite diagnosis was not confirmed.

**Fig. 1:**
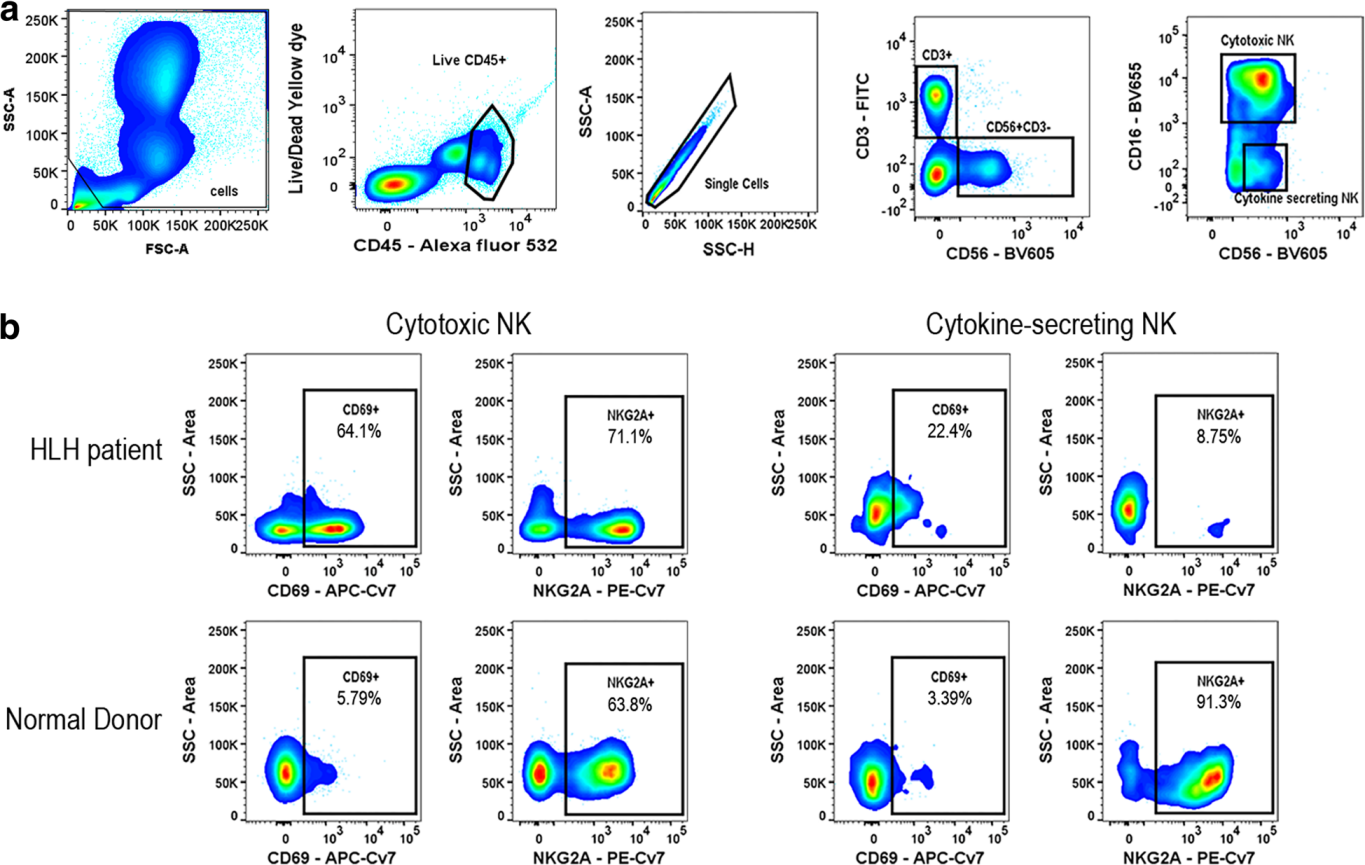
Natural killer cell flow cytometric analysis of peripheral blood mononuclear cells of patient with hemophagocytic lymphohistiocytosis. Natural killer cell gating was performed on live single CD56+ cells (**a**). Representation of CD69 and NKG2A surface expression in cytotoxic (CD56+ CD16+) and cytokine-secreting (CD56+ CD16-) natural killer cells (**b**). Results from the patient with hemophagocytic lymphohistiocytosis and a normal donor are shown. *HLH* hemophagocytic lymphohistiocytosis, *NK* natural killer

As an attempt to expedite the evaluation of possible HLH, we isolated mononuclear cells from peripheral blood and evaluated expression of surface markers in cytokine-producing NK cells and cytotoxic NK cells by flow cytometry. We compared the profile with normal controls. The results, available after 36 hours, were remarkable for an increased expression of CD69 in cytotoxic NK cells, and decreased NKG2A in cytokine-producing NK cells in our case. The expression of CD69 and NKG2A in NK cells was evaluated in four other normal donors and the results were similar to the one acquired in parallel to the HLH sample (Fig. 1). No differences in protein expression of other markers were observed by flow cytometry (data not shown). These findings included similar surface levels of OX40, GITR, 4-1BB, TIM-3, PD-1, CTLA-4, LAG-3, and ICOS in CD8+ CD3+ T cells, as well as effector (CD127+, FoxP3-) and regulatory (CD127-, FoxP3+) CD4+ CD3+ T cells; similar expression of NKp44, NKG2C, NKG2D, 4-1BB, NKp30, and NKp46 in NK cells (CD56+ CD3-); and similar expression of CD28, CD27, ICOS, Eomes, Blimp-1, Bcl-6, T-bet, Ki-67, and cMyc in naïve (CCR7+ CD45RA+), effector (CCR7- CD45RA+), effector memory (CCR7-CD45RA-), and central memory (CCR7+ CD45RA-) CD4+ and CD8+ T cells. The frequency of all the evaluated immune cell populations was also similar, when comparing cells from our patient with those ones from a healthy control.

Our patient’s bone marrow biopsy was performed 10 days after admission and delayed due to severe coagulopathy and demonstrated normocellularity (40 to 50%), megakaryocytic hypoplasia, and clusters of foamy histiocytes with ingested marrow cells. Fifteen days after admission, he fulfilled multiple HLH-2004 diagnostic criteria including hyperferritinemia, fever, splenomegaly, cytopenias, hemophagocytosis on biopsy of bone marrow, elevated CD25, and decreased NK cell activity by standardized studies (Table 1).

He steadily recovered with the steroid therapy instituted for 12 days and supportive measures. He was weaned off intubation and sedation. However, he refused further interventions for HLH, namely etoposide-based therapy and continuation of steroid treatment; he chose palliative care and died 2 weeks later.

## Discussion

Adult onset HLH is mainly a secondary phenomenon, arising from four components of pathology: a susceptible host; intrinsic/acquired immune defects; a trigger resulting in immune activation; and immunopathology resulting in host end organ failure [3]. Adult patients with HLH are immunocompromised individuals with malignancy, infection, or autoimmune disorders. Ramos-Casals *et al*. [4] found that 48% of cases were triggered by neoplasms, especially hematologic malignancies. Furthermore, patients with active malignancy are at greatest risk [4–6]. Differentiation of HLH from other sequelae of hematologic malignancies is based upon degree of inflammation [7]. Allen and McClain hypothesized that the syndrome results from complex interactions of host genetics and extrinsic immune challenges [7]. Studies have shown that immune defects in genes affecting regulation of granule-dependent lymphocyte activity as well as intracellular granule trafficking, mutations in T cell functioning (CD27), and cytokine production, that is, interferon (IFN)-gamma and interleukin (IL)-6, by malignant cells play a key role in HLH evolution [5, 8]. Similar genes and inflammatory responses may become affected during neoplasm therapy, inciting an iatrogenic HLH in adults [9–11]. The HLH-2004 criteria, validated in the pediatric community, are consensus criteria developed to guide inclusion and enrollment to studies [7]. The sensitivity and specificity of these criteria in the adult population have yet to be validated through prospective studies. However, a recent study from the MD Anderson Cancer Center (MDACC) identified an “extended 18-point diagnostic criteria” of clinical and laboratory variables closely associated with a diagnosis of malignancy-associated HLH (M-HLH) in adults. Sensitivity analysis suggested that individuals with an underlying malignancy who met 5/18 criteria could be considered to have a high likelihood of M-HLH [6]. These extended criteria include available parameters at the community level such as serum albumin, serum transaminases, lactate dehydrogenase, and basic coagulation testing that may function as early effective surrogates of M-HLH. These 18 markers are currently being validated in a prospective clinical trial at MDACC (NCT02385110).

HLH consensus definitions implicate specific cell lines responsible for the severe inflammatory propagation, including: CD8+ T lymphocytes overactivation and uncontrolled expansion and NK cell dysfunction. Familial HLH (FHL) studies identified genetic deficiencies in these cell lines [3, 7, 12]. More recently, flow cytometry has been used to screen genetically predisposed patients, measuring intracellular perforin levels in NK cells [13]. To the best of our knowledge, similar flow cytometry studies, as described in our case, have not been conducted in adult patients with secondary HLH. Although well documented in patients with malignancies, NK cell line alterations may ultimately lead to secondary HLH. Various mechanisms of NK cell anti-tumor activity inhibition have been described, including: downregulation of NK-activating receptors, that is, natural cytotoxic receptors (NCRs) and NKG2D; upregulation of inhibitory receptors, that is, killer-cell immunoglobulin-like receptors (KIRs) and NKG2A; and modifications of expression of receptor-specific ligands. Ultimately, this inhibits NK cell-mediated anti-tumor surveillance, and impairs molecular crosstalk between NK cells and other immune cells [14]. Another study noted that excessive CD8+ T cells activation during FHL suppressed the regulatory T cell (Treg) population, allowing for rapid growth of CD8+ T-cell line and unchecked progression of persistent systemic inflammation. This resulted in an overall reduction of IL-2 and reversal in the IL-2 hierarchy of consumption by T cells, with pathologic preferential consumption by inflammatory CD8+ T cells via upregulated CD25, release of sCD25 by CD8+ T cells, reduction in Treg cell IL-2-induced anti-inflammatory properties, and reduction in Treg cell lines; this ultimately resulted in diverting “the normally anti-inflammatory IL-2 feedback loop into a proinflammatory circuit” [13].

Another major problem is the lack of effective therapy. Hurdles to therapy initiation, similar to our case report, include: (a) delayed recognition of HLH, (b) advanced stage of underlying disease, and (c) concurrent immunocompromised state with a need for further cytotoxic HLH-directed therapy. However, our experience with adult HLH suggests that early suspicion and treatment are of paramount priority as high mortality rate is secondary to the aggressive and rapid HLH process [6]. Emerging non-cytotoxic therapies such as IFN-gamma inhibitor (NI-0501), anti-IL-6 agents (tocilizumab), and JAK-inhibitors (ruxolitinib), either as single agents or in combination with traditional HLH therapies, hold promise for the therapy of M-HLH in adults.

## Conclusions

The current diagnostic criteria of HLH might be suboptimal for adult patients and perhaps result in delays in its diagnosis and timely initiation of treatment. Given the high prevalence of cancer in an ageing population, current diagnostic criteria have to be re-evaluated in adult patients and novel, easily available, and highly specific diagnostic methods must be developed.
